# Efficacy and adverse effects of peripheral nerve blocks and local infiltration anesthesia after arthroscopic shoulder surgery: A Bayesian network meta-analysis

**DOI:** 10.3389/fmed.2022.1032253

**Published:** 2022-11-10

**Authors:** Zheng Liu, Yi-bo Li, Ji-hua Wang, Guang-han Wu, Peng-cai Shi

**Affiliations:** ^1^Department of Anesthesiology, The First Affiliated Hospital of Shandong First Medical University, Jinan, China; ^2^Huaiyin District Center for Disease Control and Prevent, Jinan, China

**Keywords:** arthroscopic shoulder surgery, pain management, nerve block, complications, Bayesian network meta-analysis

## Abstract

**Study objective:**

To quantitatively assess and compare the efficacy and adverse effects of six different peripheral nerve block techniques after arthroscopic shoulder surgery (ASS).

**Design:**

Bayesian network meta-analysis.

**Methods:**

The PubMed, Embase, Web of Science, the Cochrane Central Register of Controlled Trials, China National Knowledge Infrastructure database, Chinese Scientific Journal database, Wan Fang databases were searched to retrieve randomized clinical trials comparing interscalene brachial plexus block, continuous interscalene brachial plexus block, supraclavicular brachial plexus block, suprascapular nerve block, combined suprascapular and axillary nerve block and local infiltration analgesia on postoperative pain, opioid consumption, and adverse effects (defined as Horner’s syndrome, dyspnea, hoarseness, vomiting, and nausea) after ASS under general anesthesia (GA). Two reviewers independently screened the literature, extracted data, and evaluated the risk of bias in the included studies.

**Results:**

A total of 1,348 articles were retrieved initially and 36 randomized clinical trials involving 3,124 patients were included in the final analysis. The network meta-analysis showed that interscalene brachial plexus block was superior in reducing pain and opioid consumption compared to the five other interventions. However, adverse effects were reduced using suprascapular nerve block and combined suprascapular and axillary nerve block compared to interscalene brachial plexus block.

**Conclusion:**

Interscalene brachial plexus block was superior in reducing pain and opioid consumption compared to other peripheral nerve blocks but had a higher frequency of adverse events.

## Introduction

Arthroscopic shoulder surgery (ASS) is a commonly used procedure for shoulder surgery with minimal invasiveness, a wide field of vision, and rapid functional recovery (1, 2). Despite the popularity of the surgery, the severe postoperative pain becomes a complication after ASS (up to 45%) that prolongs the patient’s recovery period and seriously affect the quality of life (3). Thus, finding a safe and effective postoperative pain regimen is crucial.

Currently, general anesthesia (GA) is combined with a regional nerve block in ASS, which reduces postoperative requirements of analgesia (4). Interscalene brachial plexus block (ISB) is one of the most reliable and commonly performed regional techniques, which has been universally considered a standard technique in postoperative pain management for ASS (5, 6). However, it often associated with a risk of complications, including epidural or subarachnoid injection, Horner’s syndrome, dyspnea, hoarseness, intravascular injection, muscle or vascular injury, pneumothorax (7). Some peripheral nerve blocks involving ISB, continuous interscalene nerve block (CISB), supraclavicular nerve block (SCB), suprascapular nerve block (SSNB), suprascapular nerve block combined with axillary nerve block (SSAX) and local infiltration anesthesia (LIA) are also recommended to provide postoperative analgesia for ASS. The ranking of them in terms of efficacy and safety is still unknown, and an excellent method to investigate this is the network meta-analysis provided that certain assumptions are fulfilled.

## Methods

This systematic review is reported according to the PRISMA declaration for Network Meta-analysis and the Cochrane Handbook for the Systematic Review of Interventions (8, 9). The study evaluated existing available data retrospectively, hence neither ethical approval nor patient consent is required.

### Search strategy

A systematic literature search was designed and conducted separately by two authors to identify relevant randomized controlled trials (RCTs) on PubMed, Embase, Web of Science, the Cochrane Central Register of Controlled Trials, China National Knowledge Infrastructure database, Chinese Scientific Journal database, Wan Fang Database, from the date of database inception to 1st June 2022. There were no restrictions on publication year, region, or language. We used Medical Subject Headings (MeSH) Emtree terms, subject headings, and free-text terms in our search strategy, mainly include: “arthroscopic shoulder surgery” “arthroscopy,” “shoulder,” “nerve block,” “regional anesthesia,” “regional block,” “local block,” “interscalene nerve block,” “suprascapular nerve block,” “supraclavicular nerve block,” “suprascapular and axillary nerve blocks,” “pain,” and “analgesia.” We performed a further examination if the paper was presented in a non-English format due to certain restrictions in language.

Additionally, we conducted a battery of recursive searches and manual retrieval for major international conferences, which were presented only with an abstract that met our eligibility criteria. All above screening records will be managed using EndNote X9 (Thomson ISI Research Soft, Philadelphia, PA, USA). The established search strategies for each database were displayed in the “Search Strategies” supplement.

### Eligibility criteria and exclusion criteria and data extraction

Inclusion criteria and exclusion criteria were determined as the priority according to PICO principle. Any study that compared the efficacy of anesthesia techniques as postoperative analgesia was thought suitable for our NMA. The inclusion and exclusion criteria were as follows. *Participants:* patients who underwent ASS under GA. *Interventions:* nerve block or regional anesthesia was administered in the operating room combined with GA. *Comparators:* interventions themselves or patients received GA alone. *Outcomes*: the primary outcome was pain scores (VAS or NRS) in the PACU or within 1 h, 2 or 4 h, 6 or 8 h, 24 h after surgery and opioids consumption in 24 h after surgery; the secondary outcomes were the incidence of adverse events. *Study design:* Only RCTs were included in this review. Exclusion criteria: contraindications to nerve block or local anesthetics, coagulopathy, neuropathy, and chronic opioid use.

Two authors (ZL and J-HW) independently identified the relevant articles. Both titles and abstracts were initially searched according to the established eligible criteria. Duplicate articles were also removed simultaneously. In addition, studies published only in abstract form without any available data were discarded. If there is disagreement, an independent reviewer (P-CS) will serve as the expert referee to ensure consensus was reached on all items. Studies were summarized into seven groups, CISB, ISB, SSNB, SCB, SSAX, LIA, control group (CG).

### Outcome measures and quality assessment

Two authors extracted relevant data from the included articles independently as follows: first author(s), year of publication, patient characteristics, sample size, type of block used, pain scores, opioids consumption, incidence of complications (Horner syndrome, dyspnea, hoarseness, vomiting, and nausea). We extracted the mean and standard deviation (SD) of pain scores and opioids consumption as continuous outcomes. As for the dichotomous data, the incidence of side effects and complications were extracted from the articles.

Two independent authors (ZL and J-HW) appraised and classified the risk of bias by using Cochrane’s risk of bias (ROB) tool. Seven assessment items were classified as low, high, or unclear rank, which included random sequence generation, allocation concealment, blinding of participants and personnel, blinding of outcome assessment, incomplete outcome data, selective outcome reporting, and “other issues” under the guidance of the guidelines of Cochran’s Handbook for Systematic Reviews of Interventions (8). The assessment of ROB was performed in Review Manager (Version 5.3). Additionally, the Grade approach was used to access the quality of evidence for each association (10).

### Data analysis

Firstly, a network plot was generated for all direct comparisons to simulate a fully connected network, and a comparison-adjusted network funnel plot for funnel plot asymmetry was applied to assess the publication bias. Both analyses were performed in STATA software, version 14.0 (Stata Corp., College Station, TX). Before performing data analyzing, we assessed the transitivity and consistency assumption carefully, which underlies NMA and concerns the validity of making indirect comparisons. The baseline characteristics of participants are described using summary characteristics for the following analysis (11–13). Based on the Bayesian network meta-analysis, a non-informative prior distribution was used to compare the six interventions (14). All the outcomes were analyzed using random-effects models *via* the Markov chain Monte Carlo (MCMC) method, which established three distinct chains with sufficient iteration (15–17). For continuous variable, we used the mean difference (MD) to pool the effect size, as well as their 95% confidence intervals. As for the incidence of side effects and complications, dichotomous data were summarized using the odds ratio (OR) and 95% confidence interval (CI) (18, 19). The surface under the cumulative ranking curve (SUCRA) was calculated to rank probability of each intervention (20). A higher SUCRA value represents the likelihood that the intervention is on the top rank or is highly effective; a SUCRA value of 0 indicates the lowest efficacy compared to other prevention (19). Convergence of iterations was assessed for each parameters using the Brooks-Gelman-Rubin method and visual analysis of trace plots. The network consistency was evaluated with the node-splitting approach, where *P*-values of less than 0.05 indicated the probability of inconsistency of the entire network frame. If necessary, another sensitivity analysis was conducted for studies (8, 16, 21, 22). The above of the Bayesian network analysis was performed using the OpenBUGS (ver. 3.2.3 rev 1012, Members of OpenBUGS Project Management Group) software.

## Results

### Baseline characteristics and quality of the included studies

**FIGURE 1:**
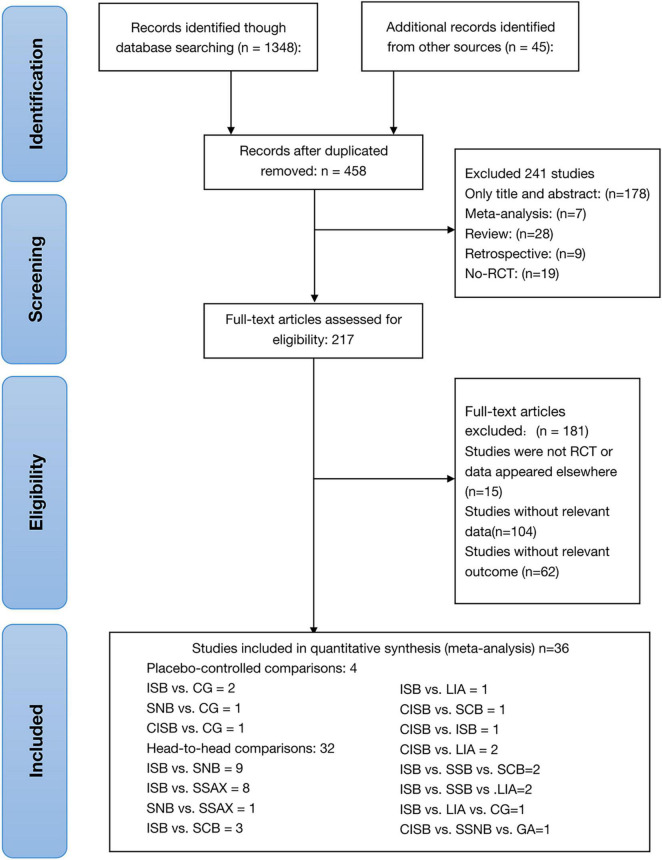
Literature review flowchart; RCT, Randomized controlled; CG, Control group.

A total of 1,348 studies were identified initially by the electronic database searches and 45 discovered by manual searching as a supplement, and 935 articles were discarded due to duplication. After screening on the titles and abstracts, 241 articles were removed, and the 217 articles that met the criteria were remained to go through a further full-text examination. 181 articles were excluded for the following reasons: 104 did not represent a relevant data, 62 did not represent a relevant outcome, 15 were not randomized controlled trials. Finally, 36 RCTs were deemed eligible for the analysis with a unanimous agreement achieved between the review authors. The outline of literature search and selection procedures are shown in Figure 1. All searched reference lists were imported and managed in EndNote X9 software (Clarivate Analytics, London, United Kingdom). The basic characteristics of included studies were summarized in Table 1.

**TABLE 1:** Characteristics of included studies.

ID	Study	Total	Age	Gender (M/F)	ASA	Primary anesthesia	Pain outcome	Ultrasound used	Amount and type of anesthetic agent	Intervention	Outcome	Complication
1	Auyong et al. (24)	189	54 ± 13 vs. 0.53 ± 14 vs. 55 ± 14	38/25 vs. 39/24 vs. 42/21	I-III	GA	NRS	Y	All: 15 mL of 0.5% ropivacaine	ISB/SSB/SCB	PACU/24 h/O	[1][2][3][4]
2	Desroches et al. (30)	53	56.5 ± 9 vs. 60.8 ± 8.7	16/9 vs. 17/11	I-III	GA	VAS	Y	ISB: 20 mL of 0.75% of ropivacaine SSB: 10 mL of 0.75% of ropivacaine	ISB/SSB	PACU/24 h	/
3	Dhir et al. (31)	59	51.3 ± 14.2 vs. 46.5 14.5	26/4 vs. 22/7	I-III	GA	NRS	N	ISB: 20 mL of 0.5% ropivacaine SSAX: 15 mL of 0.5% ropivacaine + 15 mL of 0.5% ropivacaine	ISB/SSAX	PACU/6–8 h/24 h/O	[4]
4	Kumara et al. (3)	60	60–18 years	not mention	I-II	GA	VAS	N	ISB:20 mL of 0.5% bupivacaine SSB:15 mL of 0.5% bupivacaine	ISB/SSB	PACU/6–8 h//2–4 h/24 h	/
5	Neuts et al. (44)	98	50 ± 10 vs. 0.51 ± 10	28/22 vs. 18/30	I-III	GA	VAS	Y	ISB:20 mL of 0.75% ropivacaine SSB:10 mL of 0.75% ropivacaine + 10 mL of 0.75% ropivacaine	ISB/SSAX	PACU/6–8 h//2–4 h/24 h	[4]
6	Ovesen et al. (45)	91	48.95 vs. 48.70 vs. 54.77 vs. 48.79	11/11 vs. 7/11 vs. 7/15 vs. 10/14	not mention	GA	VAS	N	ISB: 30 mL of 0.75% ropivacaine SSB: 20 mL of 0.5% bupivacaine LIA:10 mL 0.5% bupivacaine and 5 ml morphine (0.4 mg/mL)	ISB/SSB/LIA/CG	PACU/2–4 h/24 h/O	[4]
7	Singelyn et al. (50)	120	52 ± 14 vs. 54 ± 15 vs. 50 ± 14 vs. 53 ± 17	15/15 vs. 12/14 vs. 11/19 vs. 12/18	I-III	GA	VAS	N	SSB: 10 mL of 0.25% bupivacaine LIA: 20 mL of 0.25% bupivacaine ISB: 20 mL of 0.25% bupivacaine	ISB/SSB/LIA/CG	PACU/2–4 h/24 h/O	[4]
8	Yao et al. (56)	80	51.1 ± 9.29 vs. 53.03 ± 8.09	17/23 vs. 19/21	I-II	GA	VAS	Y	ISB: 20 mL of 0.5% ropivacaine SSAX: 10 mL of 0.5% ropivacaine + 10 mL of 0.5% ropivacaine	ISB/SSAX	PACU/6–8 h/24 h	[1][2][3][4]
9	Qianqian (42)	40	52.35 ± 11.90 vs. 49.55 ± 13.54 vs. 0.48.63 ± 12.68	13/20 vs. 9/20 vs. 11/20	I-II	GA	VAS	Y	ISB: 20 mL of 0.25% ropivacaine SSAX: 15 mL of 0.25% ropivacaine + 5 mL of 0.25% ropivacaine	ISB/SSAX	PACU/6–8 h//2–4 h/24 h/O	[4]
10	Pani et al. (47)	72	37.70 ± 13.65 vs. 37.06 ± 12.52	29/8 vs. 29/6	I-III	GA	VAS	Y	ISB: 10 mL of 0.75% ropivacaine SSAX: 10 mL of 0.75% ropivacaine + 10 mL of 0.75% ropivacaine	ISB/SSAX	PACU/6–8 h//2–4 h/24 h	[1][2][3][4]
11	Saini et al. (48)	70	26.97 ± 7.67 vs. 27.29 ± 6.41	31/4 vs. 0.30/5	I-II	GA	VAS	Y	ISB: 10 mL of 0.5% ropivacaine SSAX: 10 mL of 0.5% ropivacaine + 10 mL of 0.5% ropivacaine	ISB/SSAX	PACU/6–8 h//2–4 h/24 h	[2]
12	Waleed (51)	60	27.37 ± 5.87 vs. 28.57 ± 6.12	19/11 vs. 20/10	I-II	GA	VAS	Y	ISB: 20 mL of levobupivacaine 0.25% SSAX: 10 mL of levobupivacaine 0.25% + 10 mL of levobupivacaine 0.25%	ISB/SSAX	PACU/6–8 h//2–4 h/24 h/O	[1][2][3][4]
13	Aksu et al. (23)	60	45.1 ± 5.87 vs. 44.2 ± 15.9 vs. 43.4 ± 13.5	13/7 vs. 12/8 vs. 13/7	I-II	GA	VAS	Y	ISB: 20 mL 0.25% bupivacaine LIA: 20 mL 0.25% bupivacaine	ISB/LIA/CG	PACU/6–8 h//2–4 h/24 h/O	/
14	Beaudet et al. (25)	60	48 ± 11 vs. 51 ± 10	8/22 vs. 16/14	I-III	GA	NRS	N	CISB: 0.25 mL/kg of 2% lidocaine + 0.25 mL/kg of 0.5% bupivacaine LIA: 0.25 mL/kg of 2% lidocaine	CISB/LIA	PACU/24 h	/
15	Contreras-Domínguez et al. (28)	47	37 ± 7 vs. 43 ± 5	14/9 vs. 15/9	I-II	GA	VAS	N	CISB: 25 mL of 0.2% ropivacaine + 2 mg of morphine + 7 mL/h of 0.0625% bupivacaine + 1 microg/mL of sufentanil IA: 25 mL of 0.2% ropivacaine	CISB/LIA	PACU/6–8 h//2–4 h/24 h	/
16	Ikemoto et al. (35)	30	54 (39–65) vs. 57 (45–69) vs. 57 (47–76)	10/5 vs. 11/4 vs. 11/4	/	GA	VAS	N	ISB: 2 mg/kg of 0.5% ropivacaine SSB: 2 mg/kg of 0.5% ropivacaine	ISB/SSB	PACU/6–8 h/24 h	/
17	Wiegel et al. (53)	329	53 ± 13 vs. 55 ± 13	98/66 vs. 106/59	I-III	GA	VAS	Y	ISB: 20 mL 0.75% of ropivacaine SSB: 10 mL 0.75% of ropivacaine	ISB/SSB	PACU/2–4 h/24 h	[1][2][3]
18	Janssen et al. (36)	82	51 ± 10 vs. 53 ± 9	19/23 vs. 18/23	I-II	GA	VAS	N	ISB: 40 mL of 1% mepivacaine	ISB/CG	PACU/24 h	[4]
19	Abdallah et al. (7)	136	40 ± 15 vs. 46 ± 15	53/16 vs. 46/21	I-III	GA	NRS	Y	ISB: 15 mL of 0.5% ropivacaine SSB: 15 mL of 0.5% ropivacaine	ISB/SSB	PACU/6–8 h/24 h/O	/
20	Jiang et al. (37)	47	56.4 ± 13.3 vs. 55.0 ± 10.7	9/15 vs. 8/15	I-II	GA	VAS	Y	ISB: 20 mL of 0.375% ropivacaine SSB: 20 mL of 0.375% ropivacaine	ISB/SSB	PACU/6–8 h/24 h/O	/
21	Shi et al. (49)	60	55.83 ± 11.6 vs. 55.26 ± 11.75	19/11 vs. 17/13	I-II	GA	VAS	Y	SSB: 15 mL of 0.5% ropivacaine	SSB/CG	PACU/6–8 h//2–4 h/24 h	/
22	Yao et al. (55)	95	54.1 ± 9.2 vs. 53.6 ± 8.6	30/18 vs. 28/19	I-II	GA	VAS	Y	ISB: 20 mL of 0.5% ropivacaine SSB: 15 mL of 0.5% ropivacaine	ISB/SSB	PACU/6-8h//2-4h/24h/O	[1][2][3][4]
23	Janssen et al. (36)	42	54.0 ± 8.0 vs. 55.8 ± 8.0	14/7 vs. 14/7	/	GA	VAS	Y	SSB: 10 mL of 0.75% ropivacaine SSAX: 10 mL of 0.75% ropivacaine + 10 mL of 0.75% ropivacaine	SSB/SSAX	PACU/6–8 h//2–4 h/24 h	/
24	Cabaton et al. (26)	103	57 (51–65) vs. 58 (54–65)	32/20 vs. 27/24	I-II	GA	VAS	Y	ISB: 20 mL of 0.5% levobupivacaine SCB: 20 mL of 0.5% levobupivacaine	ISB/SCB	PACU/24 h/O	/
25	Karaman et al. (38)	60	52 ± 20 vs. 55.8 ± 8.0	20/11 vs. 14/15	I-II	GA	VAS	Y	ISB: 20 mL of 0.25% bupivacaine SCB: 20 mL of 0.25% bupivacaine	ISB/SCB	5 min/6–8 h/24 h	[1][2][3]
26	Koltka et al. (41)	50	48.8 ± 11.2 vs. 52.2 ± 9.8	17/8 vs. 16/9	I-II	GA	VAS	Y	ISB: 30 mL of 0.5% bupivacaine SCB: 30 mL of 0.5% bupivacaine	ISB/SCB	PACU/6–8 h//2–4 h/24 h/O	[1][3][4]
27	Wiesmann et al. (54)	114	53 ± 13 vs. 52.7 ± 13	34/22 vs. 34/24	I-II	GA	NRS	Y	ALL: 10 mL of ropivacaine 0.2% + a patient controlled analgesia (PCA) bolus of 4 ml/h 0.2% ropivacaine	CISB/SCB	PACU/24 h	[1][2][3]
28	Wang and Lin (52)	120	53 ± 12 vs. 52 ± 14 vs. 54 ± 14	24/16 vs. 25/15 vs. 27/13	I-III	GA	VAS	Y	ISB: 15 ml of 0.375% ropivacaine SSB: 15 ml of 0.375% ropivacaine	ISB/SSB/SCB	PACU/24 h/O	[1][2][3][4]
29	Faiz et al. (32)	80	48.80 ± 7.48 vs. 49.70 ± 7.05	28/12 vs. 30/10	I-II	GA	VAS	Y	ISB: 15 ml of 0.2% ropivacaine SSAX: 10 ml of 0.2% ropivacaine + 10 ml of 0.2% ropivacaine	ISB/SSAX	PACU/6–8 h/24 h/O	[4]
30	Debnath et al. (29)	105	44 (24–70) vs. 44.5 (23–73)	30/22 vs. 30/23	I-III	GA	VAS	N	ISB: 20 ml 0.5% Chirocaine LIA: 20 ml 0.5% Chirocaine	ISB/LIA	PACU/2–4 h/24 h/O	/
31	Kim et al. (40)	93	62.39 ± 8.78 vs. 59.09 ± 7.5 vs. 62.74 ± 6.92	14/17 vs. 17/14 vs. 15/16	I-II	GA	VAS	N	ISB: 15 ml 2% lidocaine + 15 ml 2% levobupivacaine SSB: Ropivacaine 10 mg + lidocaine10 mg PCA: lidocaine 100 mg + Ropivacaine 100 mg	CISB/SSB/CG	PACU/6–8 h/24 h	/
32	Gurger and Ozer (33)	85	58.47 ± 7.18 vs. 58.21 ± 7.67	25/18 vs. 22/20	I-II	GA	VAS	N	CISB: 30 ml of 0.25% bupivacaine + 5 ml/h 0.125% bupivacaine	CISB/CG	PACU/6–8 h/24 h	/
33	Kim et al. (39)	117	63.70 ± 8.13 vs. 60.78 ± 9.38 vs. 60.90 ± 9.15	17/22 vs. 19/18 vs. 17/22	I-III	GA	VAS	N	ISB: 16 ml of 0.75% ropivacaine + 4 ml of 2% lidocaine CISB: 10 ml bolus solution of 0.75% ropivacaine	CISB/ISB/CG	PACU/24 h	/
34	Cao and Yan (27)	50	57.72 ± 7.31 vs. 56.80 ± 7.34	15/10 vs. 10/15	/	GA	VAS	Y	ISB: 20 ml of 0.2% ropivacaine SSB: 20 ml of 0.2% ropivacaine	ISB/SSB	PACU/6–8 h//2–4 h/24 h	/
35	Liu (46)	107	≥ 18	/	I-III	GA	VAS	Y	ISB: 6 ml 0.3%ropivacaine SSB: 6 ml 0.3%ropivacaine	ISB/SSB	PACU/6–8 h/24 h	[2][3][4]
36	Huang and Luo (34)	60	46.3 ± 10.2 vs. 46.6 ± 10.3	13/17 vs. 11/19	I-III	GA	VAS	Y	ISB: 20 ml of 0.5% ropivacaine	ISB/CG	PACU/6–8 h/24 h	/

ASA, American Society of Anesthesiologists; PCA, Patient controlled analgesia; PACU, Post anesthesia care unit; GA, General anesthesia; VAS, Visual analog scale; NRS, numerical rating scale; O, Opioids consumption; [1], Horner syndrome; [2], Dyspnea; [3], Hoarseness; [4], Vomiting and nausea.

**FIGURE 2:**
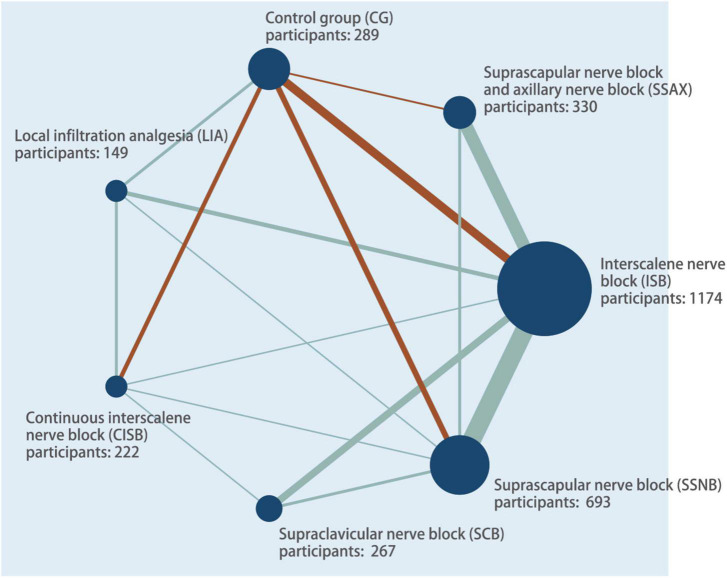
Network plot of all evidence of all the trails. The network plot of the intervention network shows the comparison of the sample size to provide anesthesia for patients undergoing arthroscopic shoulder surgery. Each node represented a different method of prevention with size of the node depending on the number of patients who received the intervention directly. The nodes were connected by lines indicating direct relationships between interventions, with the thickness of the line depending on the amount of direct evidence supporting the intervention.

Thirty-six studies included in the review were published between 2004 and 2021, enrolling a total of 3,124 patients undergoing ASS for arthroscopic rotator cuff, subacromial decompression and other forms of shoulder surgery (3, 7, 23–56). The RCTs had a parallel (*n* = 4) or crossover (*n* = 32) design between six interventions. The sample size was largest for the ISB group (*n* = 1,174; 29 studies), followed by the SSNB group (*n* = 693; 17 studies), the CISB group (*n* = 415; 7 studies), SSAX group (*n* = 330; 10 studies), and control group (*n* = 289; 9 studies), the SCB group (*n* = 267; 6 studies), and LIA group (*n* = 149; 5 studies). A network plot was generated to visualize all direct comparisons (Figure 2).

**FIGURE 3:**
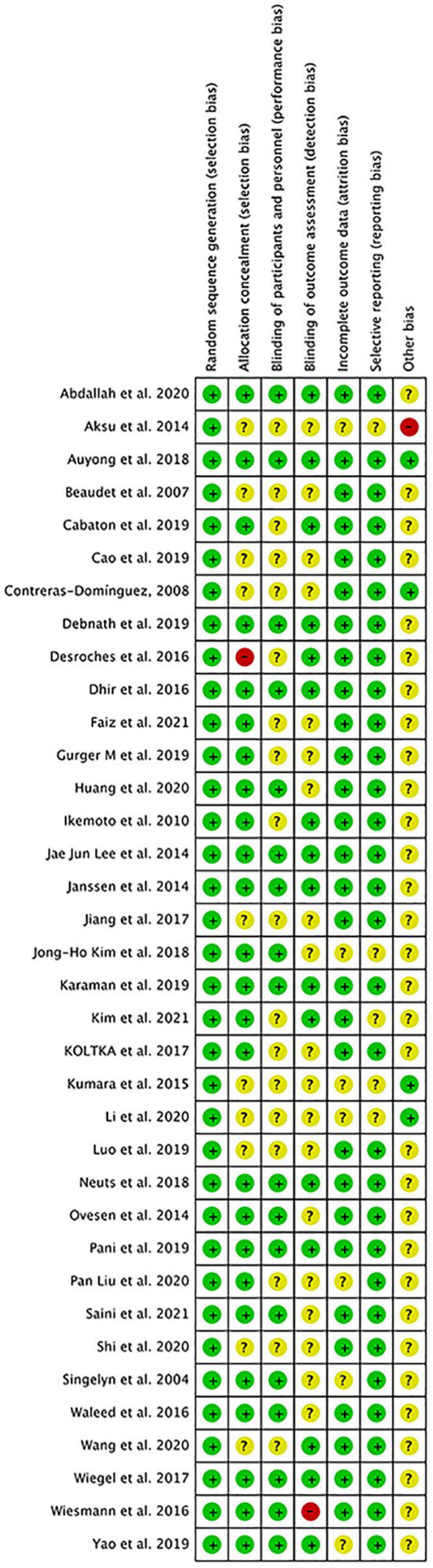
Risk of bias graph.

**FIGURE 4:**
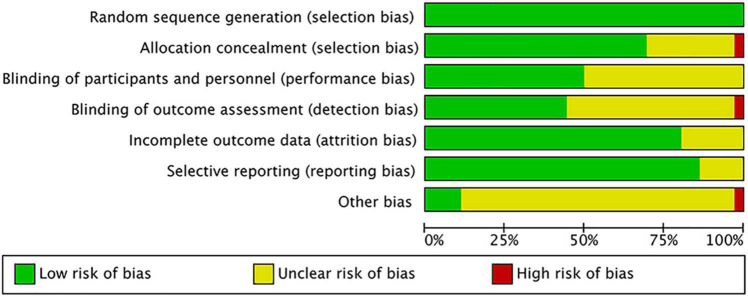
Risk of bias summary.

**FIGURE 5:**
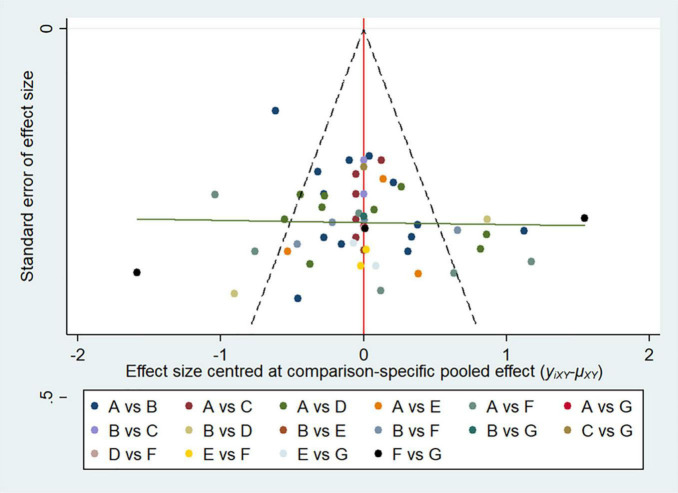
Funnel plot.

The overall quality of included studies showed low variations. All the 36 included trials were randomly assigned and had a low risk of bias (ROB) in “Random sequence generation.” Five studies had a low ROB for the selective reporting item. Seven RCTs had a high or unclear ROB due to attrition. 25 used allocation concealment and 16 described the blinding of outcome assessment in detail. The assessment of quality of included studies were showed in Figures 3, 4. The funnel plot did not indicate publication bias due to its symmetrical distribution (Inverted funnel plot) (Figure 5).

### Pain scores

Every study of postoperative pain scores has been associated with various nerve blocks or local analgesia. Thirty-one studies evaluated pain score by recording on a visual analog scale (VAS), a continuous scale based on a 0–10 cm (100 mm) in length. Five studies evaluated postoperative pain scores with a numerical rating scale (NRS) scoring, and the numbers (0–10) were administered in a numeric version of the VAS to evaluate pain intensity. The pain scores were evaluated at five time points (In the PACU or within 1 h after surgery, 2 or 4 h, 6 or 8 h, 24 h after surgery).

#### In the Post anesthesia care unit or within 1 h after surgery

A total of 36 studies reported pain scores in the PACU or within 1 h after surgery, including 7 groups (CG, ISB, CISB, SSNB, SCB, SSAX, LIA). CISB (*MD* = –3.14, 95% CI –4.47, –1.82), ISB (*MD* = –2.41, 95% CI –3.40, –1.41), SCB (*MD* = –2.34, 95% CI –3.79, –0.88), SSNB (*MD* = –1.66, 95% CI –2.73, –0.59), and SSAX (*MD* = –1.63, 95% CI –2.86, –0.39), provided significantly better analgesic effects compared to the CG group.

According to the SUCRA data (Supplementary Figure 1), CISB (SUCRA = 94.27%) and ISB (75.49%) had the highest efficacy, followed by SCB (69.36%), SSNB (39.64%), SSAX (38.79%), SSAX (31.18%), and control group (1.28%).

#### Within 2 or 4 h after surgery

Sixteen studies reported pain scores within 2 or 4 h after surgery and included 7 groups (CG, ISB, CISB, SSNB, SCB, SSAX, IA). ISB (MD = –2.02, 95% CI –3.49, –0.58) has significantly better outcomes than the CG group within 2 or 4 h after surgery.

According to the SUCRA data (Supplementary Figure 2), ISB (SUCRA = 85.56%) had the highest efficacy, followed by SCB (72.74%), SSNB (52.16%), CISB (48.53%), SSAX (48.23%), LIA (31.85%), and control group (10.92%).

#### Within 6 or 8 h after surgery

Twenty-three studies reported pain scores within 6 or 8 h after surgery and included 7 groups (Control group, ISB, CISB, SSNB, SCB, SSAX, LIA). ISB (*MD* = –1.69, 95% CI –2.54, –0.88), SCB (*MD* = –1.78, 95% CI –3.33, –0.24), SSNB (*MD* = –1.49, 95% CI –2.37, –0.63), CISB (*MD* = –1.39, 95% CI –2.50, –0.29) have significantly better outcomes than the CG group within 6 h or 8 h after surgery.

According to the SUCRA data (Supplementary Figure 3), ISB (SUCRA = 77.35%) had the highest efficacy, followed by SCB (75.37%), SSNB (62.93%), CISB (57.89%), LIA (47.02%), SSAX (27.53%), and control group (1.92%).

#### At 24 h after surgery

Thirty-six studies reported pain scores at 24 h after surgery and included 7 groups (Control group, ISB, CISB, SSNB, SCB, SSAX, LIA). SSNB (*MD* = –1.26, 95% CI –2.39, –0.10), SSAX (*MD* = –1.10, 95% CI –2.06, –0.11) have significantly better outcomes than the LIA group at 24 h after surgery.

The SUCRA data denoted that SSNB (SUCRA = 86.73%) and SSAX (SUCRA = 78.21%) had the highest efficacy, followed by ISB (SUCRA = 60.05%), CISB (SUCRA = 50.21%), SCB (SUCRA = 45.38%), LIA (SUCRA = 8.26%), and control group (21.16%) (Supplementary Figure 4).

### Opioids consumption

Eighteen studies reported opioids consumption within 24 h after surgery and included 7 groups (Control group, ISB, CISB, SSNB, SCB, SSAX, LIA). ISB (*MD* = –12.9, 95% CI –17.15, –7.08), SCB (*MD* = –8.36, 95% CI –15.48, –1.33), SSNB (*MD* = –7.15, 95% CI –12.20, –2.15) have significantly better outcomes than the CG group within 6 h or 8 h after surgery (Supplementary Figure 5).

The SUCRA data showed that ISB (SUCRA = 97.23%) had the highest efficacy, followed by, SCB (SUCRA = 67.41%), SSNB (SUCRA = 57.91%), SSAX (SUCRA = 50.76%), CISB (SUCRA = 46.85%), LIA (SUCRA = 25.71%), and control group (21.16%).

### Postoperative complications

#### Horner syndrome

Ten studies reported the incidence of Horner syndrome after surgery and included 5 groups (ISB, SSNB, SCB, CISB, SSAX). SSNB (OR = 0.15, 95% CI 0.01, 0.29), and SSAX (OR = 0.86, 95% CI 0.01, 0.67) significantly reduced the incidence of Horner syndrome compared to CISB group. SSNB (OR = 0.04, 95% CI 0.01, 0.13), SSAX (OR = 0.08, 95% CI 0.01, 0.32), and SCB (OR = 0.24, 95% CI 0.06, 0.58) significantly reduced the incidence of Horner syndrome compared to ISB group (Supplementary Figure 6).

#### Dyspnea

Twelve studies reported the incidence of dyspnea after surgery and included 5 groups (ISB, SSNB, SCB, CISB, SSAX). SSAX (OR = 0.12, 95% CI 0.02, 0.32) and SSNB (OR = 0.27, 95% CI 0.07, 0.62) significantly reduced the incidence of dyspnea syndrome compared to ISB group (Supplementary Figure 7).

#### Hoarseness

Eleven studies reported the incidence of hoarseness after surgery and included 5 groups (ISB, SSNB, SCB, CISB, SSAX). SSAX (OR = 0.29, 95% CI 0.03, 0.88) and SSNB (OR = 0.36, 95% CI 0.08, 0.84) significantly reduced the incidence of *hoarseness* compared to ISB group (Supplementary Figure 8).

#### Vomiting and nausea

Fourteen studies reported the incidence of vomiting after surgery and included 5 groups (ISB, SSNB, SCB, CISB, SSAX). SSNB (OR = 0.31, 95% CI 0.11, 0.71) and ISB (OR = 0.31, 95% CI 0.71, 0.84) significantly reduced the incidence of Horner syndrome compared to CISB group (Supplementary Figure 9).

## Discussion

This NMA provides efficacy data on five variants of nerve blocks and intra-articular infiltration analgesia combined with GA, as well as the comparisons of some important complications. In the included study, all patients received nerve block before surgery. During the perioperative period, patients received GA with muscle relaxants, combined with multimodal analgesia. It is suggested that ISB are the most highly effective performed regional techniques for ASS in the early postoperative period (in the PACU or 1 h after surgery, 2 or 4 h, 6 or 8 h), while SSNB, SSAX provided provide better late postoperative shoulder analgesia (at 24 h after surgery). Moreover, SSNB, SSNB, SCB, may have a lower overall complication rate for Horner syndrome, dyspnea, hoarseness, vomiting and nausea than ISB and CISB.

ISB has been historically considered the gold standard in postoperative pain management for ASS, which was usually performed with an injection of local anesthetic at the nerve root level of the brachial plexus to block C5–7 between the anterior and middle scalene muscles (5, 57, 58). A systematic review by Warrender et al. recommend the use of ISBs as the most effective analgesic for outpatient undergoing ASS based on the evidence of 40 RCTs (4). Consistent with previous studies, our results also indicated that ISB significantly improved pain control in the early postoperative period compared with control group, particularly in the PACU or within 2 h or 4 h hours postoperatively. Following ISB, ipsilateral phrenic nerve block is a well-known complication, of which the rates of 16.6–38% have been reported in previous studies. The root cause is the interscalene insertion site is close to the phrenic nerve, and the unintended spread of local anesthesia could cause diaphragm paresis, thus reducing vital capacity and leading to dyspnea (59). Therefore, ISB would have been a relative contraindication in patients with serious pulmonary disease. Desai found that patients who received continuous interscalene infusion catheters (CISB) resulted in a clinically remarkable improvement during the first 24 postoperative hours compared with those who received a single shot ISB (5). It is indicated in our results CISB group provided a better analgesia than the ISB group in the early postoperative period.

Many studies suggested SSNB may be considered as an alternative when ISB is contraindicated to be used as an option for patients after ASS (60–62). A previous meta-analysis of 14 articles suggested that, SSNB showed inferior analgesic effect compared with ISB, particularly in the short-term period (in the PACU or within 1–2 h postoperatively) (2). At 24 h postoperative, there was no significant difference in analgesic effect between the SSNB and ISB groups. The results of this NMA are mostly consistent with previous systematic reviews. In the early postoperative time (in PACU or within 1 h), compared to the control group, the efficiency of the SSNB group was lower than that of the ISB group (ISB: *MD* = –2.41, 95% CI –3.40, –1.41; SSNB: *MD* = –1.66, 95% CI –2.73, –0.59). Additionally, compared to the ISB group, the SSNB group provided a lower analgesic effect than the ISB group (*MD* = –0.74, 95% CI –1.48, –0.01). At 24 h after surgery, the analgesic effect has no significant difference between two groups. The explanation for the imperfect early pain control of SSNB is that, the suprascapular nerve is considered to innervate about 70% shoulder joint, the other 30% is innervated by the lateral thoracic nerve and axillary nerve (2, 63). Therefore, we hypothesize that combined with axillary block, SSAX may provide improved postoperative pain control compared with SSNB alone. The results suggested that SSAX group significantly reduced pain scores compared with control group (in PACU or at 24 h) (64). However, there was no difference between the results of SSNB group and the SSAX group. Furthermore, in contrast to that of ISB, we find that the complication rates were significantly lower in the SSNB and SSAX groups.

Supraclavicular block (SCB) is also an alternative to ISB with a low incidence of side effects. Cornish found that although SCB were administered under the clavicle and above the first rib, the local anesthetics could spread cephalad between the anterior and middle scalene muscles (65). A meta-analysis by Guo et al. compared SCB with ISB in pain control after shoulder surgery, indicating that SCB provided similar analgesic efficacy compared to ISB with a low incidence of hoarseness and Horner syndrome (66), which is consistent with our results. Compared with control group, SCB group reduced significantly pain scores in PACU (*MD* = –2.34, 95% CI –3.79, –0.88).

Local infiltration analgesia (LIA) is a safe and valuable postoperative pain management technique for patients undergoing ASS, which was usually performed at the end of the shoulder surgery before wound closure. However, iatrogenic chondrolysis of the glenohumeral joint as a complication of local infiltration analgesia is a rare but recognized complication, especially in the case of high dose and long-term administration of bupivacaine (67). In our NMA, the results suggested that LIA play no significant role in reducing the pain score at all time periods.

Our study has several strengths. To our knowledge, this is the first network meta-analysis evaluating postoperative pain regimens after ASS. Additionally, high-quality meta-analysis could be performed owing to that only RCTs was eligible for the present analysis. The trials were generally at low risk of bias for most ROB domains. Furthermore, in order to guarantee an accurate and thorough evaluation of the total body of data, the GRADE approach was used to grade the quality of the studies. Our NMA provided comprehensive evidence-based clinical practice guidance regarding the perioperative pain regimens in patients undergoing ASS.

There are also potential limitations in this review. Due to the limitations of the literature, some new analgesic methods and rare complications of nerve block were not analyzed in this NMA. Moreover, different types, concentrations, volumes of local anesthesia were used in these trials, which may cause some deviations. Another limitation is related to the technology used. Some nerve blocks are performed under ultrasound guidance, while others are located only by nerve stimulation. Furthermore, there was heterogeneity between the included studies in terms of quality evaluation, outcome measures, and assessment time. Finally, the proficiency of the operators, postoperative analgesia used, and patient characteristics may affect the pooled results and occurrence of complications.

## Conclusion

ISB was superior in reducing pain and opioid consumption compared to other peripheral nerve blocks but had a higher frequency of adverse events.

## Data availability statement

The original contributions presented in this study are included in the article/Supplementary material, further inquiries can be directed to the corresponding author.

## Author contributions

ZL and J-hW helped substantial contributions to the conception or design of the work, the acquisition, analysis, interpretation of data for the work, drafting the manuscript, and revising it critically for important intellectual content. Y-bL and G-hW helped agreement to be accountable for all aspects of the work in ensuring that questions related to the accuracy or integrity of any part of the work are appropriately investigated and resolved. P-cS helped final approval of the version to be published. All authors contributed to the article and approved the submitted version.
