# Supplementary material for: Efficacy and adverse effects of peripheral nerve blocks and local infiltration anesthesia after arthroscopic shoulder surgery: A Bayesian network meta-analysis
Source: Front Med (Lausanne). 2022 Nov 10;9:1032253. doi: 10.3389/fmed.2022.1032253 (PMC9684667; doi:10.3389/fmed.2022.1032253)
Supplement: Supplementary file 1 [file Data_Sheet_1.PDF]

The confidence in MD for efficacy at prophylaxis by GRADE system.

Comparison	Study limitation	Imprecision	Inconsistency	Indirectness	Publication bias	GRADE
CISB vs. CG	No downgrade	Upgrade due to large effect, MD= -3.14 95%CrI: -4.47 to -1.82	No downgrade	The overall effect was significantly based on the primary outcome.	Undetectable by routine method. The comparison-adjusted funnel plot for the network is not suggestive of any dominant publication bias	Moderate
ISB vs. CG	No downgrade	Upgrade due to large effect, MD= -2.41 95%CrI: -3.40 to -1.4	No downgrade	The overall effect was significantly based on the primary outcome.	Undetectable by routine method. The comparison-adjusted funnel plot for the network is not suggestive of any dominant publication bias	Moderate
SCB vs. CG	No downgrade	Upgrade due to large effect, MD= -2.34 95%CrI: -3.79 to -0.88	No downgrade	The overall effect was significantly based on the primary outcome.	Undetectable by routine method. The comparison-adjusted funnel plot for the network is not suggestive of any dominant publication bias	Moderate
SSNB vs.CG	No downgrade	No downgrade, MD=-1.66 95%CrI: -2.73 to -0.59	No downgrade	The overall effect was significantly based on the primary outcome.	Undetectable by routine method. The comparison-adjusted funnel plot for the network is not suggestive of any dominant publication bias	Low
SSAX vs. CG	No downgrade	No downgrade, MD= -1.63 95%CrI: -2.86 to -0.39	No downgrade	The overall effect was significantly based on the primary outcome.	Undetectable by routine method. The comparison-adjusted funnel plot for the network is not suggestive of any dominant publication bias	Low
LIA vs. CG	No downgrade	No downgrade, MD= -1.30 95%CrI: -3.01 to 0.43	No downgrade	The treatment effect was not significantly based on the primary outcome	Undetectable by routine method. The comparison-adjusted funnel plot for the network is not suggestive of any dominant publication bias	Low
CISB vs. LIA	No downgrade	No downgrade, MD= -1.85 95%CrI: -3.46 to -0.26	No downgrade	The treatment effect was significantly based on the primary outcome	Undetectable by routine method. The comparison-adjusted funnel plot for the network is not suggestive of any dominant publication bias	Low
ISB vs. LIA	No downgrade	No downgrade, MD= -1.11 95%CrI: -2.77 to 0.52	No downgrade	The treatment effect was not significantly based on the primary outcome	Undetectable by routine method. The comparison-adjusted funnel plot for the network is not suggestive of any dominant publication bias	Low
SCB vs. LIA	No downgrade	No downgrade, MD= -1.04 95%CrI: -2.98 to 0.87	No downgrade	The treatment effect was not significantly based on the primary outcome	Undetectable by routine method. The comparison-adjusted funnel plot for the network is not suggestive of any dominant publication bias	Low
SSNB vs. LIA	No downgrade	No downgrade, MD= -0.37 95%CrI: -2.11 to 1.36	No downgrade	The treatment effect was not significantly based on the primary outcome	Undetectable by routine method. The comparison-adjusted funnel plot for the network is not suggestive of any dominant publication bias	Low
SSAX vs. LIA	No downgrade	No downgrade, MD= -0.33 95%CrI: -2.16 to 1.49	No downgrade	The treatment effect was not significantly based on the primary outcome	Undetectable by routine method. The comparison-adjusted funnel plot for the network is not suggestive of any dominant publication bias	Low
CISB vs. SSAX	No downgrade	No downgrade, MD= -1.52 95%CrI: -3.06 to 0.02	No downgrade	The treatment effect was not significantly based on the primary outcome	Undetectable by routine method. The comparison-adjusted funnel plot for the network is not suggestive of any dominant publication bias	Low
ISB vs. SSAX	No downgrade	No downgrade, MD= -0.78 95%CrI: -1.65 to 0.08	No downgrade	The treatment effect was not significantly based on the primary outcome	Undetectable by routine method. The comparison-adjusted funnel plot for the network is not suggestive of any dominant publication bias	Low
SCB vs. SSAX	No downgrade	No downgrade, MD= -0.71 95%CrI: -2.15 to 0.72	No downgrade	The treatment effect was not significantly based on the primary outcome	Undetectable by routine method. The comparison-adjusted funnel plot for the network is not suggestive of any dominant publication bias	Low

SSNB vs. SSAX	No downgrade	No downgrade, MD= -0.04 95%CrI: -1.08 to 0.99	No downgrade	The treatment effect was not significantly based on the primary outcome	Undetectable by routine method. The comparison-adjusted funnel plot for the network is not suggestive of any dominant publication bias	Low
CISB vs. SSNB	No downgrade	No downgrade, MD= -1.48 95%CrI: -2.87 to -0.08	No downgrade	The treatment effect was significantly based on the primary outcome	Undetectable by routine method. The comparison-adjusted funnel plot for the network is not suggestive of any dominant publication bias	Low
ISB vs. SSNB	No downgrade	No downgrade, MD= -0.74 95%CrI: -1.48 to -0.01	No downgrade	The treatment effect was significantly based on the primary outcome	Undetectable by routine method. The comparison-adjusted funnel plot for the network is not suggestive of any dominant publication bias	Low
SCB vs. SSNB	No downgrade	No downgrade, MD= -0.68 95%CrI: -1.96 to 0.60	No downgrade	The treatment effect was not significantly based on the primary outcome	Undetectable by routine method. The comparison-adjusted funnel plot for the network is not suggestive of any dominant publication bias	Low
CISB vs. SCB	No downgrade	No downgrade, MD= -0.80 95%CrI: -2.38 to 0.77	No downgrade	The treatment effect was not significantly based on the primary outcome	Undetectable by routine method. The comparison-adjusted funnel plot for the network is not suggestive of any dominant publication bias	Low
ISB vs. SCB	No downgrade	No downgrade, MD= -0.07 95%CrI: -1.23 to 1.09	No downgrade	The treatment effect was not significantly based on the primary outcome	Undetectable by routine method. The comparison-adjusted funnel plot for the network is not suggestive of any dominant publication bias	Low
CISB vs. ISB	No downgrade	No downgrade, MD= -0.74 95%CrI: -2.06 to 0.59	No downgrade	The treatment effect was not significantly based on the primary outcome	Undetectable by routine method. The comparison-adjusted funnel plot for the network is not suggestive of any dominant publication bias	Low
ISB vs. LIA	No downgrade	No downgrade, MD= -2.02 95%CrI: -3.49 to -0.58	No downgrade	The treatment effect was significantly based on the primary outcome	Undetectable by routine method. The comparison-adjusted funnel plot for the network is not suggestive of any dominant publication bias	Moderate
SCB vs. LIA	No downgrade	No downgrade, MD= -2.02 95%CrI: -5.09 to 1.09	No downgrade	The treatment effect was not significantly based on the primary outcome	Undetectable by routine method. The comparison-adjusted funnel plot for the network is not suggestive of any dominant publication bias	Low
SSNB vs. LIA	No downgrade	No downgrade, MD= -1.21 95%CrI: -2.77 to 0.34	No downgrade	The treatment effect was not significantly based on the primary outcome	Undetectable by routine method. The comparison-adjusted funnel plot for the network is not suggestive of any dominant publication bias	Low
CISB vs. LIA	No downgrade	No downgrade, MD= -1.09 95%CrI: -4.10 to 1.89	No downgrade	The treatment effect was not significantly based on the primary outcome	Undetectable by routine method. The comparison-adjusted funnel plot for the network is not suggestive of any dominant publication bias	Low
SSAX vs. LIA	No downgrade	No downgrade, MD= -1.13 95%CrI: -2.93 to 0.65	No downgrade	The treatment effect was not significantly based on the primary outcome	Undetectable by routine method. The comparison-adjusted funnel plot for the network is not suggestive of any dominant publication bias	Low
CG vs. LIA	No downgrade	No downgrade, MD= -0.69 95%CrI: -2.23 to 0.84	No downgrade	The treatment effect was not significantly based on the primary outcome	Undetectable by routine method. The comparison-adjusted funnel plot for the network is not suggestive of any dominant publication bias	Low
ISB vs. CG	No downgrade	No downgrade, MD= -1.33 95%CrI: -2.60 to -0.07	No downgrade	The treatment effect was not significantly based on the primary outcome	Undetectable by routine method. The comparison-adjusted funnel plot for the network is not suggestive of any dominant publication bias	Low
SCB vs. CG	No downgrade	No downgrade, MD= -1.33 95%CrI: -4.33 to 1.69	No downgrade	The treatment effect was not significantly based on the primary outcome	Undetectable by routine method. The comparison-adjusted funnel plot for the network is not suggestive of any dominant publication bias	Low

SSNB vs. CG	No downgrade	No downgrade, MD= -0.52 95%CrI: -1.93 to 0.91	Only one head-to-head study, and no heterogeneity. One direct comparison and no -node_x005f [illegible]	The treatment effect was not significantly based on the primary outcome	Undetectable by routine method. The comparison-adjusted funnel plot for the network is not suggestive of any dominant publication bias	Low
CISB vs. CG	No downgrade	No downgrade, MD= -0.40 95%CrI: -2.99 to 2.16	No downgrade	The treatment effect was not significantly based on the primary outcome	Undetectable by routine method. The comparison-adjusted funnel plot for the network is not suggestive of any dominant publication bias	Low
SSAX vs. CG	No downgrade	No downgrade, MD= -0.44 95%CrI: -2.09 to 1.20	No downgrade	The treatment effect was not significantly based on the primary outcome	Undetectable by routine method. The comparison-adjusted funnel plot for the network is not suggestive of any dominant publication bias	Low
ISB vs. SSAX	No downgrade	No downgrade, MD= -0.89 95%CrI: -1.98 to 0.20	No downgrade	The treatment effect was not significantly based on the primary outcome	Undetectable by routine method. The comparison-adjusted funnel plot for the network is not suggestive of any dominant publication bias	Low
SCB vs. SSAX	No downgrade	No downgrade, MD= -0.89 95%CrI: -3.82 to 2.07	No downgrade	The treatment effect was not significantly based on the primary outcome	Undetectable by routine method. The comparison-adjusted funnel plot for the network is not suggestive of any dominant publication bias	Low
SSNB vs. SSAX	No downgrade	No downgrade, MD= -0.08 95%CrI: -1.43 to 1.29	No downgrade	The treatment effect was not significantly based on the primary outcome	Undetectable by routine method. The comparison-adjusted funnel plot for the network is not suggestive of any dominant publication bias	Low
CISB vs. SSAX	No downgrade	No downgrade, MD= 0.04 95%CrI: -3.01 to 3.09	No downgrade	The treatment effect was not significantly based on the primary outcome	Undetectable by routine method. The comparison-adjusted funnel plot for the network is not suggestive of any dominant publication bias	Low
ISB vs. CISB	No downgrade	No downgrade, MD= -0.93 95%CrI: -3.80 to 1.95	No downgrade	The treatment effect was not significantly based on the primary outcome	Undetectable by routine method. The comparison-adjusted funnel plot for the network is not suggestive of any dominant publication bias	Low
SCB vs. CISB	No downgrade	No downgrade, MD= -0.93 95%CrI: -4.88 to 3.04	No downgrade	The treatment effect was not significantly based on the primary outcome	Undetectable by routine method. The comparison-adjusted funnel plot for the network is not suggestive of any dominant publication bias	Low
SSNB vs. CISB	No downgrade	No downgrade, MD= -0.12 95%CrI: -3.05 to 2.83	No downgrade	The treatment effect was not significantly based on the primary outcome	Undetectable by routine method. The comparison-adjusted funnel plot for the network is not suggestive of any dominant publication bias	Low
ISB vs. SSNB	No downgrade	No downgrade, MD= -0.81 95%CrI: -1.81 to 0.17	No downgrade	The treatment effect was not significantly based on the primary outcome	Undetectable by routine method. The comparison-adjusted funnel plot for the network is not suggestive of any dominant publication bias	Low
SCB vs. SSNB	No downgrade	No downgrade, MD= -0.81 95%CrI: -3.71 to 2.09	No downgrade	The treatment effect was not significantly based on the primary outcome	Undetectable by routine method. The comparison-adjusted funnel plot for the network is not suggestive of any dominant publication bias	Low
ISB vs. SCB	No downgrade	No downgrade, MD= -0.01 95%CrI: -2.74 to 2.71	No downgrade	The treatment effect was not significantly based on the primary outcome	Undetectable by routine method. The comparison-adjusted funnel plot for the network is not suggestive of any dominant publication bias	Low
ISB vs.CG	No downgrade	Upgrade due to large effect, MD=-1.69 95%CrI: -2.54 to -0.88	No downgrade	The treatment effect was significantly based on the primary outcome	Undetectable by routine method. The comparison-adjusted funnel plot for the network is not suggestive of any dominant publication bias	Moderate
SCB vs.CG	No downgrade	Upgrade due to large effect, MD=-1.78 95%CrI: -3.33 to -0.24	No downgrade	The treatment effect was significantly based on the primary outcome	Undetectable by routine method. The comparison-adjusted funnel plot for the network is not suggestive of any dominant publication bias	Moderate

SSNB vs.CG	No downgrade	Upgrade due to large effect, MD=-1.49 95%CrI: -2.37 to -0.63	No downgrade	The treatment effect was significantly based on the primary outcome	Undetectable by routine method. The comparison-adjusted funnel plot for the network is not suggestive of any dominant publication bias	Moderate
CISB vs.CG	No downgrade	Upgrade due to large effect, MD=-1.39 95%CrI: -2.50 to -0.29	No downgrade	The treatment effect was significantly based on the primary outcome	Undetectable by routine method. The comparison-adjusted funnel plot for the network is not suggestive of any dominant publication bias	Moderate
LIA vs.CG	No downgrade	No downgrade, MD=-1.18 95%CrI: -2.56 to 0.19	No downgrade	The treatment effect was not significantly based on the primary outcome	Undetectable by routine method. The comparison-adjusted funnel plot for the network is not suggestive of any dominant publication bias	Low
SSAX vs.CG	No downgrade	No downgrade, MD= -0.84 95%CrI: -1.86 to 0.17	No downgrade	The treatment effect was not significantly based on the primary outcome	Undetectable by routine method. The comparison-adjusted funnel plot for the network is not suggestive of any dominant publication bias	Low
CISB vs.CG	No downgrade	No downgrade, MD=0.19 95%CrI: 0.10 to 0.37	No downgrade	The treatment effect was not significantly based on the primary outcome	Undetectable by routine method. The comparison-adjusted funnel plot for the network is not suggestive of any dominant publication bias	Low
ISB vs.SSAX	No downgrade	No downgrade, MD=-0.86 95%CrI: -1.51 to -0.2	No downgrade	The treatment effect was not significantly based on the primary outcome	Undetectable by routine method. The comparison-adjusted funnel plot for the network is not suggestive of any dominant publication bias	Low
SCB vs.SSAX	No downgrade	No downgrade, MD=-0.95 95%CrI: -2.40 to 0.53	No downgrade	The treatment effect was not significantly based on the primary outcome	Undetectable by routine method. The comparison-adjusted funnel plot for the network is not suggestive of any dominant publication bias	Low
SSNB vs.SSAX	No downgrade	No downgrade, MD=-0.66 95%CrI: -1.56 to 0.25	No downgrade	The treatment effect was not significantly based on the primary outcome	Undetectable by routine method. The comparison-adjusted funnel plot for the network is not suggestive of any dominant publication bias	Low
CISB vs.SSAX	No downgrade	No downgrade, MD=-0.55 95%CrI: -1.92 to 0.81	No downgrade	The treatment effect was not significantly based on the primary outcome	Undetectable by routine method. The comparison-adjusted funnel plot for the network is not suggestive of any dominant publication bias	Low
LIA vs.SSAX	No downgrade	No downgrade, MD=-0.34 95%CrI: -1.88 to 1.20	Only one head-to-head study, and no heterogeneity. One direct comparison and no -node_x005f [illegible]	The treatment effect was not significantly based on the primary outcome	Undetectable by routine method. The comparison-adjusted funnel plot for the network is not suggestive of any dominant publication bias	Low
ISB vs.LIA	No downgrade	No downgrade, MD=-0.89 95%CrI:-1.94 to 0.89	No downgrade	The treatment effect was not significantly based on the primary outcome	Undetectable by routine method. The comparison-adjusted funnel plot for the network is not suggestive of any dominant publication bias	Low
SCB vs.LIA	No downgrade	No downgrade, MD=-0.60 95%CrI: -2.53 to 1.33	No downgrade	The treatment effect was not significantly based on the primary outcome	Undetectable by routine method. The comparison-adjusted funnel plot for the network is not suggestive of any dominant publication bias	Low
SSNB vs.LIA	No downgrade	No downgrade, MD=-0.31 95%CrI: -1.77 to 1.14	No downgrade	The treatment effect was not significantly based on the primary outcome	Undetectable by routine method. The comparison-adjusted funnel plot for the network is not suggestive of any dominant publication bias	Low
CISB vs.LIA	No downgrade	No downgrade, MD=-0.21 95%CrI: -1.54 to 1.12	No downgrade	The treatment effect was not significantly based on the primary outcome	Undetectable by routine method. The comparison-adjusted funnel plot for the network is not suggestive of any dominant publication bias	Moderate
ISB vs.CISB	No downgrade	No downgrade, MD=-0.31 95%CrI: -1.54 to 0.93	No downgrade	The treatment effect was not significantly based on the primary outcome	Undetectable by routine method. The comparison-adjusted funnel plot for the network is not suggestive of any dominant publication bias	Moderate

SCB vs. CISB	No downgrade	No downgrade, MD=-0.39 95%CrI: -2.17 to 1.42	No downgrade	The treatment effect was not significantly based on the primary outcome	Undetectable by routine method. The comparison-adjusted funnel plot for the network is not suggestive of any dominant publication bias	Moderate
SSNB vs. CISB	No downgrade	No downgrade MD=-0.11 95%CrI: -1.32 to 1.11	No downgrade	The treatment effect was not significantly based on the primary outcome	Undetectable by routine method. The comparison-adjusted funnel plot for the network is not suggestive of any dominant publication bias	Low
ISB vs. SSNB	No downgrade	No downgrade, MD=-0.20 95%CrI: -0.85 to 0.45	No downgrade	The treatment effect was not significantly based on the primary outcome	Undetectable by routine method. The comparison-adjusted funnel plot for the network is not suggestive of any dominant publication bias	Low
SCB vs. SSNB	No downgrade	No downgrade, MD=-0.29 95%CrI: -1.74 to 1.19	No downgrade	The treatment effect was not significantly based on the primary outcome	Undetectable by routine method. The comparison-adjusted funnel plot for the network is not suggestive of any dominant publication bias	Low
ISB vs. SCB	No downgrade	No downgrade, MD=0.09 95%CrI: -1.24 to 1.38	No downgrade	The treatment effect was not significantly based on the primary outcome	Undetectable by routine method. The comparison-adjusted funnel plot for the network is not suggestive of any dominant publication bias	Low
SSNB vs. LIA	No downgrade	Upgrade due to large effect, MD=-1.26 95%CrI: -2.39 to -0.10	No downgrade	The treatment effect was not significantly based on the primary outcome	Undetectable by routine method. The comparison-adjusted funnel plot for the network is not suggestive of any dominant publication bias	Low
SSAX vs. LIA	No downgrade	Upgrade due to large effect, MD=-1.10 95%CrI: -2.06 to -0.11	No downgrade	The treatment effect was not significantly based on the primary outcome	Undetectable by routine method. The comparison-adjusted funnel plot for the network is not suggestive of any dominant publication bias	Low
CISB vs. LIA	No downgrade	No downgrade, MD=-0.87 95%CrI: -1.90 to 0.18	No downgrade	The treatment effect was not significantly based on the primary outcome	Undetectable by routine method. The comparison-adjusted funnel plot for the network is not suggestive of any dominant publication bias	Low
ISB vs. LIA	No downgrade	No downgrade, MD=-0.86 95%CrI: -1.76 to 0.06	No downgrade	The treatment effect was not significantly based on the primary outcome	Undetectable by routine method. The comparison-adjusted funnel plot for the network is not suggestive of any dominant publication bias	Moderate
SCB vs. LIA	No downgrade	No downgrade, MD=-0.67 95%CrI: -1.85 to 0.54	No downgrade	The treatment effect was not significantly based on the primary outcome	Undetectable by routine method. The comparison-adjusted funnel plot for the network is not suggestive of any dominant publication bias	Low
CG vs. LIA	No downgrade	No downgrade, MD=-0.32 95%CrI: -0.65 to 1.28	No downgrade	The treatment effect was not significantly based on the primary outcome	Undetectable by routine method. The comparison-adjusted funnel plot for the network is not suggestive of any dominant publication bias	Low
SSNB vs. CG	No downgrade	No downgrade, MD=-0.94 95%CrI: -1.95 to 0.08	No downgrade	The treatment effect was not significantly based on the primary outcome	Undetectable by routine method. The comparison-adjusted funnel plot for the network is not suggestive of any dominant publication bias	Low
SSAX vs. CG	No downgrade	No downgrade, MD=-0.78 95%CrI: -1.56 to 0.01	No downgrade	The treatment effect was not significantly based on the primary outcome	Undetectable by routine method. The comparison-adjusted funnel plot for the network is not suggestive of any dominant publication bias	Low
CISB vs. CG	Downgrade due to concerns about Blinding of outcome assessors	No downgrade, MD=-0.55 95%CrI: -1.49 to 0.41	No downgrade	The treatment effect was not significantly based on the primary outcome	Undetectable by routine method. The comparison-adjusted funnel plot for the network is not suggestive of any dominant publication bias	Very low (Downgrade due to study limitations)
ISB vs. CG	No downgrade	No downgrade, MD=-0.54 95%CrI: -1.27 to 0.21	No downgrade	The treatment effect was not significantly based on the primary outcome	Undetectable by routine method. The comparison-adjusted funnel plot for the network is not suggestive of any dominant publication bias	Low

SCB vs. CG	No downgrade	No downgrade, MD=-0.35 95%CrI: -1.42 to 0.73	No downgrade	The treatment effect was not significantly based on the primary outcome	Undetectable by routine method. The comparison-adjusted funnel plot for the network is not suggestive of any dominant publication bias	Low
SSNB vs. SCB	No downgrade	No downgrade, MD=-0.59 95%CrI: -1.71 to 0.49	No downgrade	The treatment effect was not significantly based on the primary outcome	Undetectable by routine method. The comparison-adjusted funnel plot for the network is not suggestive of any dominant publication bias	Low
SSAX vs. SCB	No downgrade	No downgrade, MD=-0.43 95%CrI: -1.38 to 0.52	No downgrade	The treatment effect was not significantly based on the primary outcome	Undetectable by routine method. The comparison-adjusted funnel plot for the network is not suggestive of any dominant publication bias	Low
CISB vs. SCB	No downgrade	No downgrade, MD=-0.20 95%CrI: -1.35 to 0.95	No downgrade	The treatment effect was not significantly based on the primary outcome	Undetectable by routine method. The comparison-adjusted funnel plot for the network is not suggestive of any dominant publication bias	Low
ISB vs. SCB	No downgrade	No downgrade, MD=-0.19 95%CrI: -1.06 to 0.68	No downgrade	The treatment effect was not significantly based on the primary outcome	Undetectable by routine method. The comparison-adjusted funnel plot for the network is not suggestive of any dominant publication bias	Low
SSNB vs. ISB	No downgrade	No downgrade, MD=-0.40 95%CrI: -1.11 to 0.31	No downgrade	The treatment effect was not significantly based on the primary outcome	Undetectable by routine method. The comparison-adjusted funnel plot for the network is not suggestive of any dominant publication bias	Low
SSAX vs. ISB	No downgrade	No downgrade, MD=-0.24 95%CrI: -0.78 to 0.31	No downgrade	The treatment effect was not significantly based on the primary outcome	Undetectable by routine method. The comparison-adjusted funnel plot for the network is not suggestive of any dominant publication bias	Low
CISB vs. ISB	No downgrade	No downgrade, MD=-0.01 95%CrI: -0.96 to 0.95	No downgrade	The treatment effect was not significantly based on the primary outcome	Undetectable by routine method. The comparison-adjusted funnel plot for the network is not suggestive of any dominant publication bias	Low
SSNB vs. CISB	No downgrade	No downgrade, MD=-0.39 95%CrI:-1.57 to 0.79	No downgrade	The treatment effect was not significantly based on the primary outcome	Undetectable by routine method. The comparison-adjusted funnel plot for the network is not suggestive of any dominant publication bias	Low
SSAX vs. CISB	No downgrade	No downgrade, MD=-0.22 95%CrI: -1.23 to 0.77	No downgrade	The treatment effect was not significantly based on the primary outcome	Undetectable by routine method. The comparison-adjusted funnel plot for the network is not suggestive of any dominant publication bias	Low
SSNB vs. SSAX	No downgrade	No downgrade, MD=-0.16 95%CrI: -1.02 to 0.70	No downgrade	The treatment effect was not significantly based on the primary outcome	Undetectable by routine method. The comparison-adjusted funnel plot for the network is not suggestive of any dominant publication bias	Low
SSAX vs. SCB	No downgrade	No downgrade, MD=-0.40 95%CrI: -1.11 to 0.37	No downgrade	The treatment effect was not significantly based on the primary outcome	Undetectable by routine method. The comparison-adjusted funnel plot for the network is not suggestive of any dominant publication bias	Low
ISB vs. CG	No downgrade	Upgrade due to large effect, MD=-12.09 95%CrI: -17.15 to -7.08	No downgrade	The treatment effect was not significantly based on the primary outcome	Undetectable by routine method. The comparison-adjusted funnel plot for the network is not suggestive of any dominant publication bias	Low
SCB vs. CG	No downgrade	Upgrade due to large effect, MD=-8.36 95%CrI: -15.48 to -1.33	No downgrade	The treatment effect was not significantly based on the primary outcome	Undetectable by routine method. The comparison-adjusted funnel plot for the network is not suggestive of any dominant publication bias	Low
SSNB vs. CG	No downgrade	Upgrade due to large effect, MD=-7.15 95%CrI: -12.20 to -2.15	No downgrade	The treatment effect was not significantly based on the primary outcome	Undetectable by routine method. The comparison-adjusted funnel plot for the network is not suggestive of any dominant publication bias	Low

SSAX vs. CG	No downgrade	Upgrade due to large effect, MD=-6.45 95%CrI: -13.82 to -0.90	No downgrade	The treatment effect was not significantly based on the primary outcome	Undetectable by routine method. The comparison-adjusted funnel plot for the network is not suggestive of any dominant publication bias	Low
CISB vs. CG	No downgrade	No downgrade, MD=-5.74 95%CrI: -14.31 to 2.82	No downgrade	The treatment effect was not significantly based on the primary outcome	Undetectable by routine method. The comparison-adjusted funnel plot for the network is not suggestive of any dominant publication bias	Low
LIA vs. CG	No downgrade	No downgrade, MD=-3.61 95%CrI: -9.19 to 1.95	No downgrade	The treatment effect was not significantly based on the primary outcome	Undetectable by routine method. The comparison-adjusted funnel plot for the network is not suggestive of any dominant publication bias	Low
ISB vs. LIA	No downgrade	No downgrade, MD=-8.48 95%CrI: -13.11 to -3.85	No downgrade	The treatment effect was not significantly based on the primary outcome	Undetectable by routine method. The comparison-adjusted funnel plot for the network is not suggestive of any dominant publication bias	Low
SCB vs. LIA	No downgrade	No downgrade, MD=-4.76 95%CrI: -11.58 to 2.11	No downgrade	The treatment effect was not significantly based on the primary outcome	Undetectable by routine method. The comparison-adjusted funnel plot for the network is not suggestive of any dominant publication bias	Low
SSNB vs. LIA	No downgrade	No downgrade, MD=-3.55 95%CrI: -8.69 to 1.63	No downgrade	The treatment effect was not significantly based on the primary outcome	Undetectable by routine method. The comparison-adjusted funnel plot for the network is not suggestive of any dominant publication bias	Low
SSAX vs. LIA	No downgrade	No downgrade, MD=-2.85 95%CrI: -9.92 to 4.22	No downgrade	The treatment effect was not significantly based on the primary outcome	Undetectable by routine method. The comparison-adjusted funnel plot for the network is not suggestive of any dominant publication bias	Low
CISB vs. LIA	No downgrade	No downgrade, MD=-2.13 95%CrI: -8.62 to 4.37	No downgrade	The treatment effect was not significantly based on the primary outcome	Undetectable by routine method. The comparison-adjusted funnel plot for the network is not suggestive of any dominant publication bias	Low
ISB vs. CISB	No downgrade	No downgrade, MD=-6.35 95%CrI: -14.31 to 1.62	No downgrade	The treatment effect was not significantly based on the primary outcome	Undetectable by routine method. The comparison-adjusted funnel plot for the network is not suggestive of any dominant publication bias	Low
SCB vs. CISB	No downgrade	No downgrade, MD=-2.63 95%CrI: -12.05 to 6.80	No downgrade	The treatment effect was not significantly based on the primary outcome	Undetectable by routine method. The comparison-adjusted funnel plot for the network is not suggestive of any dominant publication bias	Low
SSNB vs. CISB	No downgrade	No downgrade, MD=-1.41 95%CrI: -9.67 to 6.89	No downgrade	The treatment effect was not significantly based on the primary outcome	Undetectable by routine method. The comparison-adjusted funnel plot for the network is not suggestive of any dominant publication bias	Low
SSAX vs. CISB	No downgrade	No downgrade, MD=-0.71 95%CrI: -10.31 to 8.86	No downgrade	The treatment effect was not significantly based on the primary outcome	Undetectable by routine method. The comparison-adjusted funnel plot for the network is not suggestive of any dominant publication bias	Low
ISB vs. SSAX	No downgrade	No downgrade, MD=-5.64 95%CrI:-10.97 to -0.30	No downgrade	The treatment effect was not significantly based on the primary outcome	Undetectable by routine method. The comparison-adjusted funnel plot for the network is not suggestive of any dominant publication bias	Low
SCB vs. SSAX	No downgrade	No downgrade, MD=-1.91 95%CrI: -9.40 to 5.61	No downgrade	The treatment effect was not significantly based on the primary outcome	Undetectable by routine method. The comparison-adjusted funnel plot for the network is not suggestive of any dominant publication bias	Low
SSNB vs. SSAX	No downgrade	No downgrade, MD=-0.70 95%CrI: -7.23 to 5.85	No downgrade	The treatment effect was not significantly based on the primary outcome	Undetectable by routine method. The comparison-adjusted funnel plot for the network is not suggestive of any dominant publication bias	Low

ISB vs. SSNB	No downgrade	No downgrade, MD=-4.94 95%CrI: -8.70 to -1.19	No downgrade	The treatment effect was not significantly based on the primary outcome	Undetectable by routine method. The comparison-adjusted funnel plot for the network is not suggestive of any dominant publication bias	Low
SCB vs. SSNB	No downgrade	No downgrade, MD=-1.21 95%CrI: -7.11 to 4.71	No downgrade	The treatment effect was not significantly based on the primary outcome	Undetectable by routine method. The comparison-adjusted funnel plot for the network is not suggestive of any dominant publication bias	Low
ISB vs. SCB	No downgrade	No downgrade, MD=-3.73 95%CrI: -9.00 to 1.53	No downgrade	The treatment effect was not significantly based on the primary outcome	Undetectable by routine method. The comparison-adjusted funnel plot for the network is not suggestive of any dominant publication bias	Low
