# Supplementary material for: Efficacy and adverse effects of peripheral nerve blocks and local infiltration anesthesia after arthroscopic shoulder surgery: A Bayesian network meta-analysis
Source: Front Med (Lausanne). 2022 Nov 10;9:1032253. doi: 10.3389/fmed.2022.1032253 (PMC9684667; doi:10.3389/fmed.2022.1032253)
Supplement: Supplementary file 2 [file Data_Sheet_2.PDF]

```
model{ # *** PROGRAM STARTS
for(i in 1:ns){ # LOOP THROUGH STUDIES
w[i,1] <- 0 # adjustment for multi-arm trials is zero for control arm
delta[i,1] <- 0 # treatment effect is zero for control arm
mu[i] ~ dnorm(0,.0001) # vague priors for all trial baselines


for (k in 1:na[i]) { # LOOP THROUGH ARMS
var[i,k] <- pow(se[i,k],2) # calculate variances
prec[i,k] <- 1/var[i,k] # set precisions

y[i,k] ~ dnorm(theta[i,k],prec[i,k]) # normal likelihood
theta[i,k] <- mu[i] + delta[i,k] # model for linear predictor
dev[i,k] <- (y[i,k]-theta[i,k])*(y[i,k]-theta[i,k])*prec[i,k] #Deviance contribution
}
resdev[i] <- sum(dev[i,1:na[i]]) # summed residual deviance contribution for this trial

for (k in 2:na[i]) { # LOOP THROUGH ARMS
delta[i,k] ~ dnorm(md[i,k],taud[i,k]) # trial-specific MD distributions
md[i,k] <- d[t[i,k]] - d[t[i,1]] + sw[i,k] # mean of treat effects distributions (with
multi-arm trial correction)
taud[i,k] <- tau *2*(k-1)/k # precision of treat effects distributions (with multi-arm
trial correction)
w[i,k] <- (delta[i,k] - d[t[i,k]] + d[t[i,1]]) # adjustment for multi-arm RCTs
sw[i,k] <- sum(w[i,1:k-1])/(k-1) # cumulative adjustment for multi-arm trials
}
}
totresdev <- sum(resdev[]) #Total Residual Deviance
d[1]<-0 # treatment effect is zero for reference treatment
for (k in 2:nt){ d[k] ~ dnorm(0,.0001) } # vague priors for treatment effects
sd ~ dunif(0,5) # vague prior for between-trial SD.
tau <- pow(sd,-2) # between-trial precision = (1/between-trial variance)
# Ranking and probabilities for treatment
for(k in 1:nt) {
order[k]<- rank(d[],k)
most.effective[k]<-equals(order[k],1)
for(j in 1:nt) {
effectiveness[k,j]<- equals(order[k],j)
cumeffectiveness[k,j]<- sum(effectiveness[k,1:j])
}
}
#SUCRA
for(k in 1:nt) {
SUCRA[k]<- sum(cumeffectiveness[k,1:(nt-1)]) /(nt-1)
```

```
}
# all MDs for each treatment level comparison
for (c in 1:(nt-1)) {
for (k in (c+1):nt) {
MD[c,k] <- (d[k]-d[c]) } }
}

# Binomial likelihood, logit link
# Random effects model for multi-arm trials
model{ # *** PROGRAM STARTS
for(i in 1:ns){ # LOOP THROUGH STUDIES
w[i,1] <- 0 # adjustment for multi-arm trials is zero for control arm
delta[i,1] <- 0 # treatment effect is zero for control arm
mu[i] ~ dnorm(0,.0001) # vague priors for all trial baselines
for (k in 1:na[i]) { # LOOP THROUGH ARMS
r[i,k] ~ dbin(p[i,k],n[i,k]) # binomial likelihood
logit(p[i,k]) <- mu[i] + delta[i,k] # model for linear predictor
rhat[i,k] <- p[i,k] * n[i,k] # expected value of the numerators
dev[i,k] <- 2 * (r[i,k] * (log(r[i,k])-log(rhat[i,k])) #Deviance contribution
+ (n[i,k]-r[i,k]) * (log(n[i,k]-r[i,k]) - log(n[i,k]-rhat[i,k])))
}
resdev[i] <- sum(dev[i,1:na[i]]) # summed residual deviance contribution for this trial
for (k in 2:na[i]) { # LOOP THROUGH ARMS
delta[i,k] ~ dnorm(md[i,k],taud[i,k]) # trial-specific LOR distributions
md[i,k] <- d[t[i,k]] - d[t[i,1]] + sw[i,k] # mean of LOR distributions (with multi-arm
trial correction)
taud[i,k] <- tau *2*(k-1)/k # precision of LOR distributions (with multi-arm trial
correction)
w[i,k] <- (delta[i,k] - d[t[i,k]] + d[t[i,1]]) # adjustment for multi-arm RCTs
sw[i,k] <- sum(w[i,1:k-1])/(k-1) # cumulative adjustment for multi-arm trials
}
}
totresdev <- sum(resdev[]) #Total Residual Deviance
d[1] <- 0 # treatment effect is zero for reference treatment
for (k in 2:nt){ d[k] ~ dnorm(0,.0001) } # vague priors for treatment effects
sd ~ dunif(0,5) # vague prior for between-trial SD.
tau <- pow(sd,-2) # between-trial precision = (1/between-trial variance)
# pairwise ORs and LORs for all possible pair-wise comparisons, if nt>2
for (c in 1:(nt-1)) {
for (k in (c+1):nt) {
or[c,k] <- exp(d[k] - d[c])
lor[c,k] <- (d[k]-d[c])
}
}
```

```
# Ranking and probabilities for treatment
for(k in 1:nt) {
order[k]<- rank(d[],k) #events are bad
#order[k]<- nt+1-rank(d[],k) #events are good
most.effective[k]<-equals(order[k],1)
for(j in 1:nt) {effectiveness[k,j]<- equals(order[k],j)
cumeffectiveness[k,j]<- sum(effectiveness[k,1:j])
}
}
#SUCRA
for(k in 1:nt) {
SUCRA[k]<- sum(cumeffectiveness[k,1:(nt-1)]) /(nt-1)
}
}
```
