# Supplementary material for: Efficacy and adverse effects of peripheral nerve blocks and local infiltration anesthesia after arthroscopic shoulder surgery: A Bayesian network meta-analysis
Source: Front Med (Lausanne). 2022 Nov 10;9:1032253. doi: 10.3389/fmed.2022.1032253 (PMC9684667; doi:10.3389/fmed.2022.1032253)
Supplement: Supplementary file 4 [file Data_Sheet_4.PDF]

Desroches, et al

	mean	val2.5pc	val97.5pc
MD[1,2]	-1.28	-3.00	0.47
MD[1,3]	-1.62	-2.86	-0.38
MD[1,4]	-1.70	-2.80	-0.63
MD[1,5]	-2.34	-3.82	-0.86
MD[1,6]	-2.39	-3.40	-1.39
MD[1,7]	-3.14	-4.49	-1.79
SUCRA[1]	1.35%	0.00	0.17
SUCRA[2]	30.81%	0.00	0.83
SUCRA[3]	38.26%	0.17	0.83
SUCRA[4]	41.09%	0.17	0.67
SUCRA[5]	69.33%	0.17	1.00
SUCRA[6]	75.02%	0.50	1.00
SUCRA[7]	94.12%	0.50	1.00

1.Placebo 2.LIA 3.SSAX 4.SSNB 5.SCB 6.ISB 7.CISB

Aksu, et al.

	mean	val2.5pc	val97.5pc
MD[1,2]	-1.36	-3.28	0.59
MD[1,3]	-1.35	-2.58	-0.13
MD[1,4]	-1.42	-2.50	-0.35
MD[1,5]	-2.08	-3.51	-0.63
MD[1,6]	-2.09	-3.11	-1.09
MD[1,7]	-3.06	-4.38	-1.73
SUCRA[1]	1.77%	0.00	0.17
SUCRA[2]	39.47%	0.00	0.83
SUCRA[3]	35.45%	0.17	0.67
SUCRA[4]	37.66%	0.17	0.67
SUCRA[5]	67.61%	0.17	1.00
SUCRA[6]	71.74%	0.50	1.00
SUCRA[7]	96.31%	0.67	1.00

1.Placebo 2.LIA 3.SSAX 4.SSNB 5.SCB 6.ISB 7.CISB

Janssen, et al.

	mean	val2.5pc	val97.5pc
MD[1,2]	-1.30	-2.98	0.38
MD[1,3]	-1.39	-2.60	-0.18
MD[1,4]	-1.79	-2.84	-0.75
MD[1,5]	-2.36	-3.76	-0.96
MD[1,6]	-2.38	-3.34	-1.43
MD[1,7]	-3.15	-4.44	-1.87
SUCRA[1]	1.29%	0.00	0.17
SUCRA[2]	31.87%	0.00	0.83

SUCRA[3]	31.09%	0.17	0.67
SUCRA[4]	45.76%	0.17	0.83
SUCRA[5]	70.49%	0.33	1.00
SUCRA[6]	74.70%	0.50	1.00
SUCRA[7]	94.80%	0.67	1.00

1.Placebo 2.LIA 3.SSAX 4.SSNB 5.SCB 6.ISB 7.CISB

Karaman, et al.

	mean	val2.5pc	val97.5pc
MD[1,2]	-1.29	-3.01	0.46
MD[1,3]	-1.63	-2.88	-0.38
MD[1,4]	-1.67	-2.76	-0.60
MD[1,5]	-2.34	-3.88	-0.79
MD[1,6]	-2.41	-3.42	-1.42
MD[1,7]	-3.14	-4.50	-1.79
SUCRA[1]	1.37%	0.00	0.17
SUCRA[2]	31.18%	0.00	0.83
SUCRA[3]	39.19%	0.17	0.83
SUCRA[4]	40.12%	0.17	0.67
SUCRA[5]	68.77%	0.17	1.00
SUCRA[6]	75.56%	0.50	1.00
SUCRA[7]	93.81%	0.50	1.00

1.Placebo 2.LIA 3.SSAX 4.SSNB 5.SCB 6.ISB 7.CISB

Koltka, et al.

	mean	val2.5pc	val97.5pc
MD[1,2]	-1.29	-3.04	0.48
MD[1,3]	-1.64	-2.89	-0.39
MD[1,4]	-1.68	-2.76	-0.59
MD[1,5]	-2.34	-3.89	-0.77
MD[1,6]	-2.42	-3.42	-1.42
MD[1,7]	-3.14	-4.50	-1.78
SUCRA[1]	1.37%	0.00	0.17
SUCRA[2]	31.08%	0.00	0.83
SUCRA[3]	39.28%	0.17	0.83
SUCRA[4]	40.29%	0.17	0.67
SUCRA[5]	68.54%	0.17	1.00
SUCRA[6]	75.67%	0.50	1.00
SUCRA[7]	93.77%	0.50	1.00

1.Placebo 2.LIA 3.SSAX 4.SSNB 5.SCB 6.ISB 7.CISB

Wiesmann, et al.

	mean	val2.5pc	val97.5pc
MD[1,2]	-1.37	-3.10	0.39

MD[1,3]	-1.59	-2.84	-0.32
MD[1,4]	-1.63	-2.72	-0.53
MD[1,5]	-2.14	-3.75	-0.54
MD[1,6]	-2.36	-3.39	-1.35
MD[1,7]	-3.31	-4.74	-1.89
SUCRA[1]	1.29%	0.00	0.17
SUCRA[2]	34.86%	0.00	0.83
SUCRA[3]	39.10%	0.17	0.83
SUCRA[4]	40.21%	0.17	0.67
SUCRA[5]	62.98%	0.17	1.00
SUCRA[6]	75.93%	0.50	1.00
SUCRA[7]	95.63%	0.67	1.00

1.Placebo 2.LIA 3.SSAX 4.SSNB 5.SCB 6.ISB 7.CISB

Wang, et al.

	mean	val2.5pc	val97.5pc
MD[1,2]	-1.30	-3.05	0.48
MD[1,3]	-1.63	-2.89	-0.37
MD[1,4]	-1.63	-2.74	-0.52
MD[1,5]	-2.42	-4.00	-0.82
MD[1,6]	-2.42	-3.44	-1.41
MD[1,7]	-3.15	-4.52	-1.78
SUCRA[1]	1.39%	0.00	0.17
SUCRA[2]	31.49%	0.00	0.83
SUCRA[3]	39.29%	0.17	0.83
SUCRA[4]	38.69%	0.17	0.67
SUCRA[5]	70.79%	0.17	1.00
SUCRA[6]	74.91%	0.50	1.00
SUCRA[7]	93.43%	0.50	1.00

1.Placebo 2.LIA 3.SSAX 4.SSNB 5.SCB 6.ISB 7.CISB

Kim, et al.

	mean	val2.5pc	val97.5pc
MD[1,2]	-1.70	-3.42	0.03
MD[1,3]	-1.71	-2.93	-0.49
MD[1,4]	-1.70	-2.82	-0.58
MD[1,5]	-2.53	-3.98	-1.08
MD[1,6]	-2.50	-3.50	-1.50
MD[1,7]	-3.84	-5.32	-2.37
SUCRA[1]	0.56%	0.00	0.17
SUCRA[2]	38.86%	0.00	0.83
SUCRA[3]	36.05%	0.17	0.67
SUCRA[4]	35.12%	0.17	0.67
SUCRA[5]	69.41%	0.17	1.00

SUCRA[6]	71.78%	0.50	0.83
SUCRA[7]	98.22%	0.83	1.00

1.Placebo 2.LIA 3.SSAX 4.SSNB 5.SCB 6.ISB 7.CISB
